# The PanCareSurFup cohort of 83,333 five-year survivors of childhood cancer: a cohort from 12 European countries

**DOI:** 10.1007/s10654-018-0370-3

**Published:** 2018-03-02

**Authors:** Desiree Grabow, Melanie Kaiser, Lars Hjorth, Julianne Byrne, Daniela Alessi, Rodrigue S. Allodji, Francesca Bagnasco, Edit Bárdi, Andrea Bautz, Chloe J. Bright, Florent de Vathaire, Elizabeth A. M. Feijen, Stanislaw Garwicz, Oskar Hagberg, Riccardo Haupt, Mike M. Hawkins, Zsuzsanna Jakab, Leontien C. M. Kremer, Claudia E. Kuehni, Rahel Kuonen, Päivi Maria Lähteenmäki, Raoul C. Reulen, Cécile M. Ronckers, Carlotta Sacerdote, Giao Vu-Bezin, Finn Wesenberg, Thomas Wiebe, David L. Winter, Jeanette Falck Winther, Lorna Zadravec Zaletel, Peter Kaatsch, on behalf of the PanCareSurFup Consortium

**Affiliations:** 1 German Childhood Cancer Registry (GCCR), Institute for Medical Biostatistics, Epidemiology and Informatics (IMBEI), University Medical Center, Johannes Gutenberg University Mainz, Mainz, Germany; 2 Department of Clinical Sciences, Skane University Hospital, Lund University, Pediatrics, Lund Sweden; 3 Boyne Research Institute, 5 Bolton Square, East, Drogheda, Co. Louth A92 RY6K Ireland; 4 Childhood Cancer Registry of Piedmont, Cancer Epidemiology Unit, Citta’ della Salute e della Scienza Hospital-University of Turin and Center for Cancer Prevention (CPO), Via Santena 7, 10126 Turin, Italy; 5 Cancer and Radiation, Unit 1018 INSERM, University Paris-Saclay, Gustave Roussy, 39, rue Camille Desmoulins, 94805 Villejuif Cedex, France; 6 Epidemiology and Biostatistics Unit, Gaslini Children’s Hospital, Via Gerolamo Gaslini, 5, 16148 Genova, Italy; 7 2nd Department of Pediatrics, Semmelweis University, Budapest, Hungary; 8 Kepler Universitätsklinikum, Linz, Austria; 9 Danish Cancer Society Research Center, Strandboulevarden 49, 2100 Copenhagen, Denmark; 10 Centre for Childhood Cancer Survivor Studies, Institute of Applied Health Research, University of Birmingham, Birmingham, B15 2TT UK; 11 Department of Pediatric Oncology, Emma Children’s Hospital/Academic Medical Center Amsterdam, Meibergdreef 9, 1105 AZ Amsterdam, The Netherlands; 12 Regional Cancer Centre South, Lund, Sweden; 13 Hungarian Childhood Cancer Registry, 2nd Department of Pediatrics, Semmelweis University, Budapest, Hungary; 14 Princess Maxima Centre for Paediatric Oncology, Utrecht, The Netherlands; 15 Swiss Childhood Cancer Registry, Institute of Social and Preventive Medicine, University of Bern, Bern, Switzerland; 16 Department of Paediatrics, University Children’s Hospital of Bern, University of Bern, Bern, Switzerland; 17 Department of Pediatric and Adolescent Medicine, Turku University and Turku University Hospital, Turku, Finland; 18 Norwegian Cancer Register Department of Pediatric Medicine, Faculty of Medicine, Oslo University Hospital and Institute of Clinical Medicine, University of Oslo, Oslo, Norway; 19 Division of Radiotherapy, Institute of Oncology, Zaloška cesta 2, 1000 Ljubljana, Slovenia

**Keywords:** European Cohort, Childhood and adolescent cancer, 5-Year survivors, Late effects, Follow-up, Epidemiology

## Abstract

Childhood cancer survivors face risks from a variety of late effects, including cardiac events, second cancers, and late mortality. The aim of the pan-European PanCare Childhood and Adolescent Cancer Survivor Care and Follow-Up Studies (PanCareSurFup) Consortium was to collect data on incidence and risk factors for these late effects among childhood cancer survivors in Europe. This paper describes the methodology of the data collection for the overall PanCareSurFup cohort and the outcome-related cohorts. In PanCareSurFup 13 data providers from 12 countries delivered data to the data centre in Mainz. Data providers used a single variable list that covered all three outcomes. After validity and plausibility checks data was provided to the outcome-specific working groups. In total, we collected data on 115,596 patients diagnosed with cancer from 1940 to 2011, of whom 83,333 had survived 5 years or more. Due to the eligibility criteria and other requirements different numbers of survivors were eligible for the analysis of each of the outcomes. Thus, 1014 patients with at least one cardiac event were identified from a cohort of 39,152 5-year survivors; for second cancers 3995 survivors developed at least one second cancer from a cohort of 71,494 individuals, and from the late mortality cohort of 79,441 who had survived at least 5 years, 9247 died subsequently. Through the close cooperation of many European countries and the establishment of one central data collection and harmonising centre, the project succeeded in generating the largest cohort of children with cancer to date.

## Introduction

Cancer in childhood is rare: for every child who contracts cancer more than 100 adults get cancer. The overall age-standardised incidence rate in Europe is 140 cases per million children aged 0–14 [1]. Currently, 80% of children diagnosed in developed countries survive to at least 5 years [2]. Unlike survivors of adult cancer survivors of childhood cancer have their whole adult lives ahead of them. The growing numbers of survivors bring increasing concern about the long-term consequences of treatment to growing organs and tissues. Each year there are approximately 35,000 new cases of cancer in young people in Europe and 1 out of 300 new-borns will develop cancer before their 20^th^ birthday [3]. At present hundreds of thousands of EU citizens have survived cancer in childhood or adolescence. It is estimated that this number will reach nearly 500,000 by 2020 [3]. At least two-thirds will have late effects caused by cancer treatment [4, 5]. In Europe, several childhood cancer-related survivor cohorts exist or are in the process of becoming established [6]. To work to achieve equity of access to care for childhood cancer survivors across Europe and to perform collaborative research PanCare—the Pan-European Network for Care of Survivors after Childhood and Adolescent Cancer—was founded in Lund, Sweden, in 2008. PanCare became a legal entity in 2013 and was granted charitable status in 2014 in the Netherlands [7]. PanCare (www.pancare.eu) is a multidisciplinary network of professionals, survivors and their families.

As part of PanCare the EU funded project PanCare-SurFup (PanCare Childhood and Adolescent Cancer Survivor Care and Follow-Up Studies; www.pancaresurfup.eu) started in 2011 as a cooperation of 16 partners [7] and was formally completed at the end of January 2017. Within PanCareSurFup (PCSF) not only partners who are funded via the EU but also a number of additional data providers (DPs) have collected data on type of cancer, cancer treatment and the occurrence of complications of cancer treatments in order to create a retrospective European cohort of more than 100,000 former childhood cancer patients. This cohort formed the basis for all the analyses carried out in the working groups of the project [7]. The work package (WP) structure was as follows: WP1 for data collection and harmonisation of data, WP2 for to collect radiation therapy data and reconstruct radiation doses to selected organs and anatomical sites, WP3 for cardiac disease, WP4 for second cancers, and WP5 for late mortality. Table 1 describes WP1 and the outcome-related work packages, WP2 to 5. The methodology for the data collection for case–control selection will be described elsewhere.

**Table 1:** Collection of data for the PanCareSurFup cohort: work packages (WP) 1–5 in PanCareSurFup: title, leader and selected objectives

WP1: Data Collection and Harmonisation (D. Grabow/P. Kaatsch, Mainz, Germany)
Establish the retrospective pan-European cohort of long-term survivors in whom one, or more, of the relevant endpoints occurred: cardiac disease, second cancer, late mortality
Provide data sets for outcome-related work packages dealing with these three endpoints
WP2: Radiation Dosimetry (F. de Vathaire, Villejuif, France)
Perform radiation therapy reconstruction and whole body dosimetry for the subjects included in WP3 and WP4 who received radiotherapy
Estimate radiation dose received to the heart during radiotherapy, as well as uncertainties in this estimate for WP3 patients
Estimate radiation dose received to the specific site of the second malignant neoplasm during radiotherapy, as well as uncertainties in this estimate for WP4 patients
WP3: Cardiac disease: cohort and nested case–control study (L. Kremer, Amsterdam, the Netherlands)
Establish a pan-European cohort of survivors of childhood cancer for whom the occurrence of cardiovascular disease has been systematically ascertained and validated in 5 years childhood cancer survivors (cases graded according the CTCAE v3 criteria; http://ctep.cancer.gov)
Determine the incidence and absolute risk of cardiovascular disease
Undertake a nested case–control study to determine aspects of radiotherapy and chemotherapy associated with increased risk of cardiac disease
WP4: Subsequent primary neoplasms: cohort and nested case–control studies (M. Hawkins, Birmingham, UK)
Compare observed and expected numbers of second primary cancers (sarcomas of bone and soft tissue and carcinomas of digestive tract and genito-urinary organs), particularly among survivors who are aged over 40 years
Undertake a nested case–control study of subsequent primary sarcomas and subsequent primary “adult-type” carcinomas as these are the most frequently observed within the cohort
Undertake those nested case–control studies to determine aspects of radiotherapy and chemotherapy most strongly associated with these subsequent primary neoplasms
WP5: Late mortality (S. Garwicz, Lund, Sweden)
Establish a pan-European cohort of survivors for which all deaths occurring at least 5 years after diagnosis and for which an official cause of death is available
Relate absolute and excess risk (compared to background population) of death from specific causes to gender, type of childhood cancer, age at diagnosis, period of cancer diagnosis and, in a subset of patients, type of treatment
Validate the official causes of death and assess the comparability and quality of causes of death in different countries

In this paper we describe the tasks and methods to establish the overall cohort of 12 European countries that formed the basis for all PanCareSurFup analyses and reports. The overall cohort of survivors will be described.

## Methods

### Data flow of data sets from 12 countries to establish the PCSF cohort

The basic cohort of survivors was established by WP1 at the University Medical Center in Mainz, Germany. WP1 had several tasks, e.g. (a) to establish the retrospective pan-European cohort of long-term survivors in whom one, or more, of the relevant endpoints occurred: cardiac disease, second cancer, late mortality, and (b) to provide data sets for “outcome-related work packages” dealing with these three endpoints for the cohort studies. The methods of WP1 are reported in this paper including data flow of data sets from 12 countries to establish the PCSF cohort, setting up the study protocol and the variable list, the requirements of the call-for-data and process of data transfer, the strategy for data protection, safety and security, and data cleaning and validity checks.

In PanCareSurFup 13 DPs from 12 countries delivered data: France, Hungary, Italy (a hospital-based and a population-based data set), the Netherlands, the Nordic Countries (Denmark, Finland, Iceland, Sweden, and Norway), Slovenia, Switzerland, and the UK (Table 2). Those DPs were either population-based cancer registry cohorts, 5-year survivor cohorts, or national clinical databases with broad coverage. Italy was an exception with two different kinds of DPs, providing data of a population-based and a clinical database setting.

**Table 2:** Characteristics of data provider (DP) contributions of the entire cohort

Country		Affiliations	Responsible contact person	Years of diagnosis (first cancer)	Year of last follow-up with respect to death	Age at last follow-up in years (percentiles)^a^		Median observation time (years)^a^
						50%	95%	
1	France	Institut Gustave Roussy (IGR), Villejuif	Florent de Vathaire	1946–1986^b^	2015	33	52	28
2	Hungary	Semmelweis University, Budapest	Zsuzsanna Jakab	1971–2009	2014	24	41	16
3	Italy (pop.based)^c^	Universita Degli Studi di Torino (Unito), Torino	Carlotta Sacerdote	1964–2009	2014	24	42	12
4	Italy (hosp.based)^d^	Italian Registry of Off therapy Patients (OTR-AIEOP), Genova	Riccardo Haupt	1960–2008	2014	27	44	20
5	The Netherlands	Academic Medical Centre, Amsterdam	Leontien Kremer	1963–2001	2013	29	46	21
6	Denmark	Danish Cancer Registry, Copenhagen	Jeanette Falck Winther	1943–2003	2006	28	53	17
7	Sweden	Swedish Cancer Register, Stockholm	Jeanette Falck Winther	1958–2003	2008	30	56	19
8	Norway	Cancer Registry of Norway, Oslo	Jeanette Falck Winther	1953–2002	2002	28	53	-^e^
9	Finland	Finnish Cancer Registry, Helsinki	Jeanette Falck Winther	1953–2011	2012	29	59	19
10	Iceland	Icelandic Cancer Society, Rykjavik	Jeanette Falck Winther	1955–2003	2008	29	54	17
11	Slovenia	Institute of Oncology, Ljubljana	Lorna Zadravec Zaletel	1960–2002	2014	32	56	24
12	Switzerland	Swiss Childhood Cancer Registry, Bern	Claudia Kuehni	1964–2005	2014	24	44	15
13	UK	The University of Birmingham, Birmingham	Mike Hawkins	1940–1991	2006	31	53	24

^a^ Based on 5-year survivor cohort

^b^ Only solid tumours

^c^ Pop. Based = population-based

^d^ Hosp. Based = hospital-based

^e^ No information about death

DPs were identified by a survey carried out by PanCare specifically for the PCSF grant application. So DPs had to fulfil specific prerequisites: (a) be able to return to the treating clinic and collect original therapy data, (b) be able to perform comprehensive follow-up of the individual survivor, and (c) capture information about at least one of the following events: prevalent cardiac events or prospective cardiac adverse events in the follow-up of the patients, second primaries, and about vital status and for deceased patients the date and cause of death, encoded in compliance with international classification systems.

**Fig. 1:**
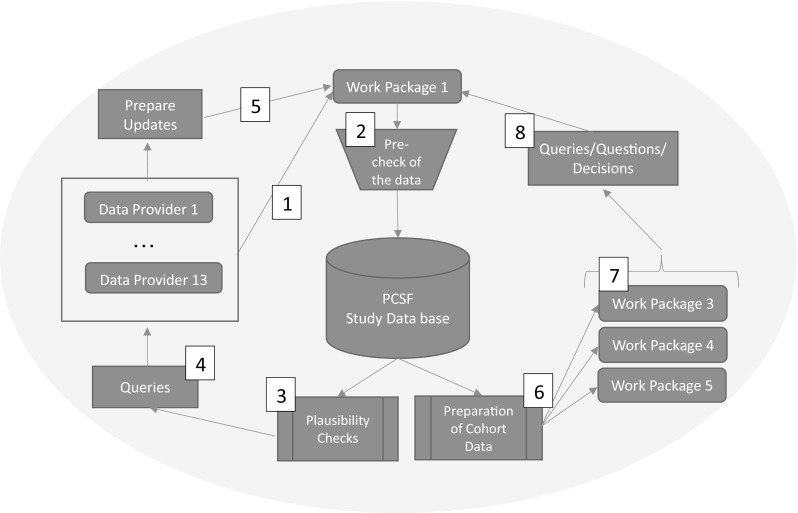
Data flow between data provider, central work package WP1 (with its PanCareSurFup study database) and three outcome-related work packages 3–5 (1: DPs delivered data; 2: WP1 checked technical quality; 3: WP1 ran plausibility checks; 4: queries to DPs to clarify implausibility; 5: DPs sent corrected data set; 6: WP1 prepared WP-specific cohorts; 7: WP1 makes WP-specific cohorts available; 8: WP sent queries to WP1)

The data flow within PanCareSurFup regarding establishment of the PCSF cohort and provision of data sets for the three outcome-related work packages 3–5 is shown in Fig. 1.

At a later stage of the project WP3 and WP4 had also to contact DPs directly, e.g. to collect details of radiotherapy and chemotherapy from the original treatment centres for the case control selection. No DP could start data collection until they received ethical approval from their ethics board within each participating country, in accordance to national laws and requirements. Additionally, an Ethical and Scientific Advisory Board was set up at the start of PCSF to support and guide the project.

### Study protocol and variable list

The participants developed the study protocol and a list of common variables. The PCSF variable list was based on variables that were already available in the databases of all DPs and could be retrieved and delivered to WP1. It was critical to establish and maintain the same standards for each WP, e.g. the list of variables was arranged particularly with regard to the three outcomes mentioned above and the same set of standards were kept for each outcome. One of the main tasks consisted in harmonising baseline variables to make them suitable for all the different study objectives. Therefore the project team early on agreed on a common set of variables. This made the work of the DPs easier as they could deliver their data for different work packages in a standard format with a common set of variables. Among others the list of 74 variables concerned the following:

- cardiac disease (13 variables): e.g. specification of all cardiac events, dates of cardiac events, dates of entry and exit from risk for cardiac disease. Cardiac events are namely symptomatic heart failure, cardiac ischemia, pericarditis, valvular disease and arrhythmia graded according to the Criteria for Adverse Events (CTCAE) [8]. More details are described in [9].
- second cancer (15 variables): e.g. number of subsequent tumours, type of each of second cancers by site, behaviour dates of entry and exit from risk for second cancer. Second cancers had to be histologically different from the first primary neoplasm and to have a malignant behaviour code. For further details see [10, 11].
- follow-up and death (18 variables): e.g. year of latest follow-up in relation to vital status, date of death, underlying and contributing causes of death. Causes of death were classified according to different versions of ICD6 to ICD10. An algorithm was developed to group all the causes of death into 14 categories. For the category “neoplasms”, patients who had more than one neoplasm registered were—at default—considered to have died from a subsequent neoplasm. To validate this assumption, data were manually scrutinized and if information was sufficient to deem that the patient actually died from the primary neoplasm, the classification was changed. In cases that remained unclear, this was clarified in a dialogue with data providers. Finally, the categories of causes of death were aggregated into several groups such as primary neoplasm, subsequent neoplasm, infection, circulatory causes, external causes, and other causes. A publication is under preparation.

This scheme of variables (available upon request) enabled a precise description of relevant data from each European country. Additionally, an exchange of data between the outcome-related work packages was possible since they had the same data structure. For instance, when the cause of death was either cardiac disease or second cancer a linkage from WP5 to WP3 and WP4 was possible.

### Call-for-data, data transfer and data safety and security

After the variable list and the common study protocol was agreed WP1 prepared and sent a formal “call-for-data”. The call-for-data specified rules regarding form and content of the data delivery, set out procedures to ensure data privacy and safety and set deadlines. Data delivery commenced at 8 May 2012 and ended on 31 December 2015. The results regarding the PanCareSurFup cohort presented here are based on the data set frozen on the 11^th^ January 2016.

The transfer of data between DP, WP1, and outcome-related WPs as well as the entire data processing in WP1 was carried out without identifying information about any individual. Another measure to protect the data was that data are exclusively transferred in encrypted form. A multi-stage security concept was designed to meet modern standards for data security and data safety to the highest degree.

### Data cleaning and validity checks

All data delivered from DPs were imported in a study database at WP1´s site. A plausibility check concept was developed. Checks were programmed with SAS 9.4 [12] and included more than 150 single check procedures. Checks dealt with the original data files delivered by the DPs (Fig. 1, step 1). In a more technical step (step 2) data were transformed in a suitable data format, which meant e.g. to restructure a wide format into different outcome related data packages and imported by VBA programmes procedures (Visual Basics for Applications, which are part of the Windows office package). The third step included checks for plausibility and validity regarding

- identification variables (unique numerical values identifying individuals within PanCareSurFup)
- valid coding (categorical variables), plausible values, proper use of the defined missing values and NULL fields
- cross-checks between “date”-variables (month and year of birth, death, last follow-up)
- counter variables for subsequent primary neoplasms and cardiac events
- special checks (e.g. age range, identifying 5-year survivors)
- checks on the ICD- and ICD-O-coding regarding causes of death and coding diagnoses. Codes referring to ICD7, 8, 9, and 10 have been accepted as well as codes referring to ICD-O-1, -2 and -3 [e.g. 13–17].
- late mortality variables (e.g. cause of death is missing)
- data regarding second cancer (e.g. regarding ICD-O and ICD-Code)
- cardiac variables (e.g. implausible or missing code of the cardiac events)

To make the distribution of different childhood cancer diagnoses comparable between the different DPs we transformed the codes into International Classification of Childhood Cancer. IARC/IACR Tools are available to transfer ICD-O into ICD-O-2 and ICD-O-3 using a conversion programme provided by the International Agency for Research on Cancer (IARC) [18]. The transition from ICD-O-2 to ICCC is going back on the IARC Child Check Program published in International Classification of Childhood Cancer (IARC Technical report No. 29, 1996). The transfer of ICD-O to ICCC3 (International Classification of Childhood Cancer, Third edition (ICCC-3) [19]) is adapted to this IARC Child Check Program and a long-term used tool developed at the German Childhood Cancer Registry, which we approved while transferring all ICD-O codes to ICCC-3 codes to allow comparability with other international sources at our registry.

There were some ICD-O-Codes included in ICD-O-1, ICD-O-2, or ICD-O-3 but diagnoses are not defined in ICCC-3. Those diagnoses are relevant diagnoses for paediatric cancer in general but had to be categorized as “further classifiable but non ICCC-3 diagnoses”, as they were benign, not further classifiable, or in situ diagnoses (e.g. Langerhans cell-histiocytosis, appendix carcinoid, lipoma, schwannoma, cavernous haemangioma, haemangioblastoma, ganglioneuroma, neurofibromatosis). As the Norwegian data set is mainly based on ICD-7 we were not able to transform these data sets into ICCC-3 appropriately (“unclassifiable”).

Following validity checks WP1 asked DPs to clarify identified implausibility (step 4) and to send a corrected data set (step 5). As a final step overall summary reports were used to discuss last remaining open issues with all WP leaders. Once all these steps were completed WP1 assembled the PanCareSurFup cohort (step 6).

## Results

### Data provision and validity checks

Data sets came from the DPs in batches. In most cases they came with updates to individual subjects. The plausibility and validity checks and the resulting questions led, in total, to more than 70 data sets delivered from the DPs to WP1, including revisions and updates from the 13 DPs to WP1 (one DP sent data 20 times, the remaining 12 DPs sent data 6 to 9 times each). Data sets included a set of additional patients or just single additional patients, event-specific additional data, or corrected files due to erroneous data. Simultaneously WP1 sent WP-specific cohort data 38 times to the three outcome-related WPs: seven data deliveries to two and 24 deliveries to the third one.

Among others, the following challenges occurred when checking the data which made further updates necessary:

- At the beginning of the cycles of plausibility checks there were more errors regarding simpler procedures like non-valid codes or incorrect time sequence.
- One DP at first provided data only for cases with an event (cardiac disease, second cancer, late mortality) but not for cases without any of these events. So data from the entire cohort was requested and received.
- The most complex errors occurred when checking the ICD- and ICD-O-coding. For example, ICD-O topography codes normally start with a ‘C’, i.e. malignant disease. A few codes sent started with a ‘D’ (non-malignant). The discussion concerning this issue led to the decision to include Langerhans cell-histiocytosis though this is not coded as malignant.

In another case, one DP delivered a large number of cases with unknown morphology and topography, respectively, based on about 100 different self-defined codes. Extensive discussion with the DP were required to discern the meaning of these codes. In a next step these codes were adapted, e.g. to ICD-O-2-topography. Many issues required discussion of individual cases for resolution. Since this DP had run out of resources, WP1 adapted the data for the DP.

### The PanCareSurFup cohort

**Fig. 2:**
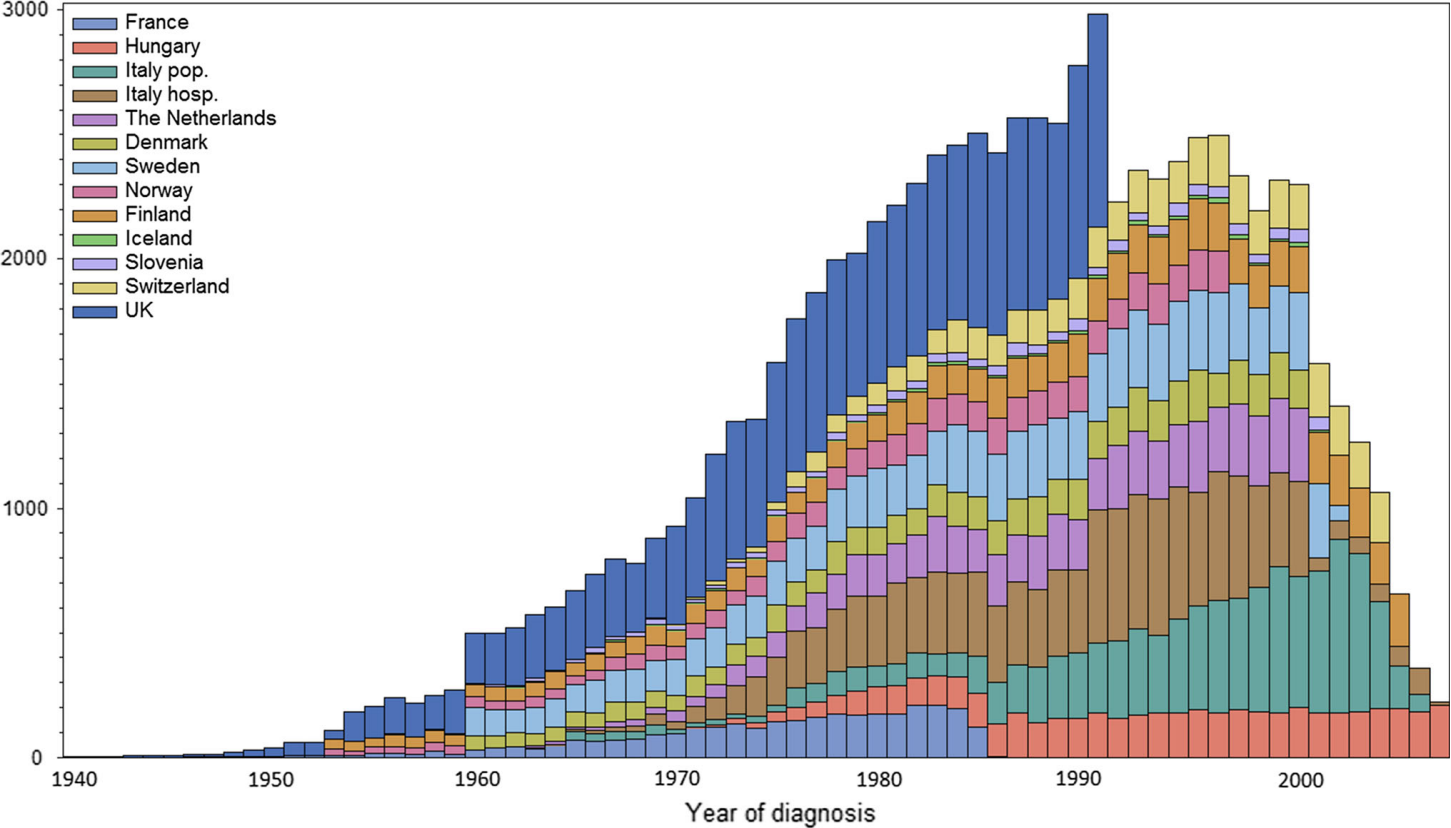
Number of cases in the PanCareSurFup 5-year survivor cohort by year of diagnosis and by data provider (based on 83,333 individuals as specified in Table 3)

The overall cohort is described divided by different DPs and with respect to length of follow-up and distribution of sex, age and diagnostic groups: This data collection effort formed the basis for WP3 to 5. Each DP included all ICCC-3 diagnostic groups with the exception of France, where only patients with solid tumours were reported. The year of diagnosis varied widely (Table 2): three DPs started in the 1940s (France, Denmark, the UK), most started in the 1950s and 1960s. End of case assessment was between 1986 (France) and 2011 (Finland). The span of years of diagnosis covered by the data from each DP varied between 38 and 60 years with the widest span in Denmark (1943–2003) and Finland (1953–2011). Figure 2 shows the distribution of cases by year of diagnosis and by data provider. Overall, most patients added to the PanCareSurFup cohort were diagnosed in the 1980s and 1990s. Some DPs added about the same number of patients each year (e.g. the Nordic Countries), while others (e.g., Italy, France and Hungary) provided more numbers in later years.

The median observation time varied between 12 and 28 years (Table 2) (based on the late mortality cohort; for the cardiac cohort and the second cancer cohort observation time was somewhat shorter). For the entire cohort the median observation time was 16 years. Follow-up ended for most DPs in the 2010s (latest follow-up year was 2015 for France), for some DPs follow-up ended at the beginning of the 2000s (Table 2). For each DP more than half of the patients were older than 23 years at date of latest follow-up; for three DPs the median was more than 30 years. Some DPs provided data sets in which more than 5% of the individuals are older than 50 years at the latest follow up (95% percentile). Of the 5-year survivor cohort with 83,333 individuals, the 50 and 95% percentile of age at latest follow-up were 28 and 51 years, respectively.

### The cohort of 5-year survivors as part of the PanCareSurFup cohort

It was expected from the beginning that the main part of the entire cohort would be the 5-year survivor cohort, described in Table 3. However, where possible, DPs were asked to provide data on their entire cohorts (i.e. all cases registered irrespective of the follow-up time). Several DPs provided only 5-year survivors (France, Hungary, The Netherlands, Switzerland and the UK). Others provided cohorts which included patients from the date of diagnosis (Nordic countries, Slovenia and Italy population based), while Italy hospital-based included only patients who had reached the elective end of therapy (off therapy), regardless of its timing with respect to the date of diagnosis. Totally, 83,333 5-year survivors were reported. Together with reported patients followed up less than 5 years (i.e. 32,263 individuals) the cohort sums up to a cohort of 115,596 patients. In the 5-year survivor cohort of 83,333 individuals, the UK and the Italian hospital-based cohort contributed more than 10,000 cases, five others contributed between 5,000 and 10,000 cases. The median follow-up time for the 5-year survivor cohort was 20 years, ranging from 12 to 28 years.

**Table 3:** Size of PanCareSurFup (PCSF) cohort and outcome specific cohorts with number of events by data provider

Country		Total number of patients included in PCSF cohort		Outcome specific 5-year survivor cohorts^a^					
				Cardiac cohort		Second cancer cohort		Late mortality cohort	
		Entire cohort	5-Year survivor cohort	No. of patients in cardiac cohort	No. of patients with at least one cardiac event	No. of patients in second cancer cohort	No. of patients with at least one second cancer	No. of patients in late mortality cohort	No. of deceased patients
1	France	3171	3146	3146	192	3157	419	3146	757
2	Hungary	5167	5142	4907	71	4920	160	5142	441
3	Italy—pop.^b^	15,124	9477	1554	57	8117	262	9477	555
4	Italy—hosp.^c^	12,315	11,051	1576	21	1576	123	11,051	976
5	The Netherlands	6087	6087	5189	176	6087	352	6087	617
6	Denmark	12,099	4822	–	–	4966	306	4822	589
7	Sweden	15,180	9302	–	–	8348	373	9302	863
8	Norway	8562	3892	–	–	3892	108	–	–
9	Finland	12,243	6341	–	–	6341	401	6341	855
10	Iceland	609	351	–	–	302	10	351	34
11	Slovenia	2341	1258	1155	21	1259	115	1258	179
12	Switzerland	4717	4483	3645	30	4549	144	4483	332
13	UK	17,981	17,981	17,980	446	17,980	1222	17,981	3049
Total		115,596^d^	83,333	39,152	1014	71,494	3995	79,441	9247

^a^ For each cohort calculated on the basis of the respective date of follow-up, but including the death date from the late mortality cohort

^b^ Pop. = population-based

^c^ Hosp. = hospital-based

^d^ A thorough investigation by WP4 of the SPN component of the entire cohort (n = 115,596) resulted in an agreed denominator of 105,015 individuals to enter into the SPN analyses

The three outcome-specific cohorts were based on the 5-year survivor cohort, as follows:

- For cardiac events (WP3) data from eight DPs were collected. Among 39,152 survivors, 1014 experienced cardiac events. Most individuals with at least one event were reported from the UK (446 patients with at least one cardiac event), France (192), and the Netherlands (176) with the remaining DPs each providing fewer than 100 cases.
- For second cancers (WP4) every DP was able to contribute data. Among 71,494 5-year survivors 3995 individuals developed at least one second cancer during the reporting period. Most individuals were reported from the UK (1222 patients with at least one second cancer), France (419), and Finland (401).
- For the late mortality cohort, data from all DPs besides Norway were available. This resulted in the cohort of 79,441 individuals of whom 9247 were deceased. Most patients deceased were reported from the UK (3049), Italy (hospital-based) (976), Sweden (863), and Finland (855).

Tables 4–6 describe the cohort of 83,333 5-year survivors by sex, age, and diagnoses. Sex ratios showed the expected male predominance with little variability (Table 4). The distribution by age at diagnosis varied as some country specific cohorts covered mostly children up to 14 years, while others covered cancer cases up to 20 years. Table 5 shows which diagnostic codes were used in the different DPs specific cohorts. Nine DPs coded diagnoses for all patients by using solely ICD-O-3 as the most current version of ICD-O. The others used older editions of ICD-O: France coded with ICD-O-1 as well as with ICD-O-2. Denmark and Sweden used ICD-O-1 or ICD-O-2, respectively. For the majority of cases in the Norwegian cohort only ICD7 was reported. Table 6 shows the distribution of cases by ICCC-3 diagnostic groups for each DP. For the sake of comparability with data from European population-based cancer registries, we included the distribution of the twelve corresponding diagnostic groups reported from ACCIS (Automated Childhood Cancer Information System project) in this table [20].

**Table 4:** PanCareSurFup (PCSF) 5-year survivor cohort by age at diagnosis and data provider

	No. of 5-year survivors PCSF cohort	Age at diagnosis			Sex ratio at time of diagnosis (male/female)
		0–14 years N (%)	15–17 years N (%)	18–20 years N (%)	
France	3146	2988 (95)	155 (4.9)	3 (0.1)	1.2
Hungary	5142	4613 (89.7)	469 (9.1)	60 (11.7)	1.3
Italy—pop.^a^	9477	6420 (67.7)	1456 (15.4)	1601 (16.9)	1.2
Italy—hosp.^b^	11,051	10,468 (94.7)	496 (4.5)	87 (0.8)	1.2
The Netherlands	6087	5565 (91.4)	489 (8.0)	33 (5.4)	1.3
Denmark	4822	3112 (64.5)	796 (16.5)	914 (19.0)	1.3
Sweden	9302	6117 (65.8)	1545 (16.6)	1640 (17.6)	1.1
Norway	3892	2441 (62.7)	636 (16.3)	815 (20.9)	1.1
Finland	6341	4158 (65.6)	1060 (16.7)	1123 (17.7)	1.0
Iceland	351	212 (60.4)	65 (18.5)	74 (21.1)	1.1
Slovenia	1258	1009 (80.2)	249 (19.8)	0 (0)	1.2
Switzerland	4483	3500 (78.1)	552 (12.3)	431 (9.6)	1.3
UK	17,981	17,450 (97.0)	531 (3.0)	0 (0)	1.2
Total	83,333	68,053 (81.7%)	8499 (10.2%)	6781 (8.1%)	

^a^ Pop. = population-based

^b^ Hosp. = hospital-based

**Table 5:** PanCareSurFup (PCSF) 5-year survivor cohort by provided classification and data provider

	ICD-7-Coding^a^	ICD-O-1^b^	ICD-O-2^b^	ICD-O-3^b^	Total
France	0	2084	1062	0	3146
Hungary	0	0	0	5142	5142
Italy—pop.^c^	0	0	0	9477	9477
Italy—hosp.^d^	0	0	0	11,051	11,051
The Netherlands	0	0	0	6087	6087
Denmark	0	4822	0	0	4822
Sweden	0	0	9291	11	9302
Norway	3103	0	789	0	3892
Finland	0	0	0	6341	6341
Iceland	0	0	0	351	351
Slovenia	0	0	0	1258	1258
Switzerland	0	0	0	4483	4483
UK	0	0	0	17,981	17,981
Total	3103	6906	11,142	62,182	83,333

^a^ ICD-7 = International Classification of Diseases, Revision *7*

^b^ ICD-O = International Classification of Diseases for Oncology, Version 1, 2, 3

^c^ Pop. = population-based

^d^ Hosp. = hospital-based

**Table 6:** PanCareSurFup (PCSF) 5-year survivor cohort transformed to ICCC-3^a^diagnostic group by data provider

		No. of 5-year survivors PCSF cohort N (%)	Group of diagnosis					
			I Leukemias N (%)	II Lymphomas N (%)	III Tumors of the central nervous system N (%)	IV Neuro-blastoma N (%)	V Retino-blastoma N (%)	VI Renal tumors N (%)
1	France	3146	–	560 (17.8)	438 (13.9)	425 (13.5)	146 (4.6)	639 (20.3)
2	Hungary	5142	1515 (29.5)	832 (16.2)	1048 (20.4)	383 (7.5)	128 (2.5)	360 (7.0)
3	Italy—pop.^d^	9477	2188 (23.1)	1961 (20.7)	1570 (16.6)	415 (4.4)	176 (1.9)	370 (3.9)
4	Italy—hosp.^e^	11,051	4591 (41.5)	1966 (17.8)	752 (6.8)	985 (8.9)	85 (0.8)	938 (8.5)
5	The Netherlands	6087	2094 (34.4)	983 (16.2)	842 (13.8)	324 (5.3)	33 (0.5)	596 (9.8)
6	Denmark	4822	912 (18.9)	692 (14.4)	1144 (23.7)	145 (3.0)	175 (3.6)	226 (4.7)
7	Sweden	9302	1640 (17.6)	1183 (12.7)	2253 (24.2)	205 (2.2)	252 (2.7)	448 (4.8)
8	Norway	3892	–	2 (0.1)	150 (3.9)	22 (0.6)	24 (0.6)	30 (0.8)
9	Finland	6341	1297 (20.5)	1023 (16.1)	1333 (21.0)	222 (3.5)	176 (2.8)	314 (5.0)
10	Iceland	351	60 (17.1)	51 (14.5)	65 (18.5)	9 (2.6)	6 (1.7)	14 (4.0)
11	Slovenia	1258	279 (22.2)	246 (19.6)	232 (18.4)	36 (2.9)	34 (2.7)	80 (6.4)
12	Switzerland	4483	1280 (28.6)	777 (17.3)	713 (15.9)	205 (4.6)	115 (2.6)	220 (4.9)
13	UK	17,981	4851 (27.0)	2307 (12.8)	4111 (22.9)	792 (4.4)	1200 (6.7)	1505 (8.4)
Total		83,333	20,707 (24.9)	12,583 (15.1)	14,651 (17.6)	4168 (5.0)	2550 (3.1)	5740 (6.9)
ACCIS Data for comparison [16]		77,111	26,690 (34.6)	8971 (11.6)	17,057 (22.1)	5580 (7.2)	1995 (2.6)	4549 (5.9)

		Group of diagnosis							
		VII Hepatic tumors N (%)	VIII Malignant bone tumors N (%)	IX Soft tissue sarcomas N (%)	X Germ cell tumors N (%)	XI Other malignant epithelial neoplasms N (%)	XII Other malignant neoplasms N (%)	Other^b^: e.g.LCH^c^ N (%)	Unclassifiable N (%)
1	France	28 (0.9)	228 (7.3)	356 (11.3)	178 (5.7)	113 (3.6)	8 (0.3)	10 (0.3)	17 (0.5)
2	Hungary	50 (1.0)	259 (5.0)	261 (5.1)	146 (2.8)	106 (2.1)	10 (0.2)	32 (0.6)	12 (0.2)
3	Italy—pop.^d^	50 (0.5)	385 (4.1)	497 (5.2)	527 (5.6)	832 (8.8)	122 (1.3)	378 (4.0)	6 (0.1)
4	Italy—hosp.^e^	65 (0.6)	285 (2.6)	623 (5.6)	238 (2.2)	78 (0.7)	26 (0.2)	417 (3.8)	2 (0.0)
5	The Netherlands	52 (0.9)	369 (6.1)	451 (7.4)	231 (3.8)	98 (1.6)	7 (0.1)	3 (0.1)	4 (0.1)
6	Denmark	19 (0.4)	160 (3.3)	278 (5.8)	436 (9.0)	484 (10.0)	43 (0.9)	71 (1.5)	37 (0.8)
7	Sweden	61 (0.7)	371 (4.0)	497 (5.3)	551 (5.9)	899 (9.7)	327 (3.5)	614 (6.6)	1 (0.0)
8	Norway	12 (0.3)	36 (0.9)	45 (1.2)	61 (1.6)	75 (1.9)	7 (0.2)	39 (1.0)	3389 (87.1)
9	Finland	33 (0.5)	265 (4.2)	384 (6.1)	342 (5.4)	784 (12.4)	59 (0.9)	107 (1.0)	2 (0.0)
10	Iceland	2 (0.6)	17 (4.8)	23 (6.7)	22 (6.3)	46 (13.1)	1 (0.3)	28 (8.0)	7 (2.0)
11	Slovenia	6 (0.5)	56 (4.5)	91 (7.2)	68 (5.4)	92 (7.3)	3 (0.2)	7 (0.6)	28 (2.2)
12	Switzerland	32 (0.7)	211 (4.7)	268 (6.0)	232 (5.2)	270 (6.0)	11 (0.3)	149 (3.3)	0 (0)
13	UK	65 (0.4)	664 (3.7)	1180 (6.7)	638 (3.4)	614 (3.4)	43 (0.2)	7 (0.0)	4 (0.0)
Total		475 (0.6)	3306 (4.0)	4954 (5.9)	3670 (4.4)	4491 (5.4)	667 (0.8)	1862 (2.2)	3509 (4.2)
ACCIS Data for comparison [16]		749 (1.0)	3692 (4.8)	5111 (6.6)	2555 (3.3)	1874 (2.4)	339 (0.4)	–	–

^a^ International Classification of Childhood Cancer, Third edition [14]

^b^ Other further classifiable diagnoses: defined in ICD-O, but not in ICCC-3

^c^ LCH = Langerhans cell-hystiocytosis

^d^ Pop. = population-based

^e^ Hosp. = hospital-based

## Discussion

The aim of WP1 in the PanCareSurFup project was to amalgamate data of survivors after childhood cancer from European cancer registries and other databases which were available for the three outcomes relevant to PanCare-SurFup (cardiac events, second cancer, late mortality). Based on this, clinical epidemiological studies were carried out on a selected set of serious late effects.

Through the cooperation of 16 project partners and 13 DPs from 12 countries, the project succeeded in generating the largest cohort of children with cancer in Europe to date. The resulting cohort of 83,333 5-year survivors is unique due to its size and the collection of a selected set of late effects. Additionally 32,263 non-five year survivors were collected, resulting in a cohort of 115,596 individuals. It provides an excellent opportunity to compare each decade since the 1940 with respect to childhood cancer and allows for a good comparison of survival rates.

Compared to other population-based European data collections, like ACCIS [1, 20, 21], some diagnoses differ in numbers, but the overall distribution in the PCSF cohort corresponds with the ACCIS data. While considering that variety of diagnoses in different countries is not uncommon to a certain extent [22] only few deviations can be seen in Table 6, primarily caused by the two further classification groups we implemented (“other further classifiable” like Langerhans cell-histiocytosis and “unclassifiable” with respect to ICCC-3). Furthermore, we have to take into account that we cannot entirely compare those two resources as ACCIS collects data since diagnosis, and our cohort is based on 5-year survivors, i.e. starts 5 years after diagnosis. Diagnoses with poorer survival (e.g. CNS tumours) were underrepresented compared to incidence data at time of diagnosis. Additionally, due to the fact that France delivered a cohort without leukaemia patients, this group contributes a little bit less than about a third to the data. Further on we seem to have a slight underreporting regarding tumours of the central nervous system (CNS), which is a known phenomenon as this diagnostic group with its different histology and behaviour is heterogeneously collected in cancer registries [23]. Neuroblastoma are somewhat less and lymphomas are somewhat more frequent compared to ACCIS. Regarding quality indicators, almost all of the data sources included in PanCareSurFup contributed as well to ACCIS, where no substantial difference between quality indicators was seen for the different data providers [24].

The assembled PanCareSurFup cohort is characterised by inclusion of all malignant diseases occurring from 0 to 20 years of age, with the exceptions previously mentioned. It should particularly be pointed out that the three outcomes relevant to PanCareSurFup are being investigated in approximately the same basic population. While cancer registries routinely collect mortality and second cancer incidence, other outcomes, such as cardiac disease, is not routine. In PCSF a small number of DPs were able to collect cardiac morbidity.

The project includes all DPs which were identified by a preceding survey and fulfil relevant requirements (e.g. good quality of follow-up, availability of relevant information, legal and organisational prerequisites). Thus, data are often collected through a population-based cancer registry, through a body with close connection to a population-based cancer registry, or within a clinical registry. In the future, statements largely representative of the population will be possible based on these analyses. Some countries that would have participated could not provide data for a variety of reasons. First, in some countries information on these outcomes was not centrally available; in other countries retrieving therapy data from clinical sources was not possible, and finally some potential DPs were uncertain that the data could be provided within the project period. The Nordic countries could not provide cardiac events due to the ongoing parallel Nordic study ALiCCS [25].

The cohort is based on data sets which were collected in very different contexts. For example, the Nordic countries had already established population-based cancer registries with high data quality and high completeness in the middle of the last century. However they lack precise information on treatment. Other countries, e.g., in Eastern Europe, also have long-standing data collections, not previously contributed to bigger projects. The persons responsible had very diverse backgrounds (epidemiologists, clinicians, registry experts) with different technical equipment and experience. DPs who were less experienced in delivering data to huge consortia received assistance from WP1 to deliver data which met the characteristics of the PCSF baseline variable list. Additionally, differences in background and level of experience were ironed out through regular meetings and bi-weekly conference calls. The use of a common data structure reduced differences between data sources.

The homogeneity of the PCSF cohort data was ensured by the following procedures: The creation of a common baseline variable list, standardised data flow and uniform data sets. All WP leaders early on determined the extent and content of the characteristics, the naming of variables, and the coding. The technical procedure of the transfer of encrypted data and the schedule for data delivery were also fixed. The call-for-data, i.e., the starting point for data delivery by the DP, included all these specifications. The harmonisation contained technical validity checks, plausibility checks, and further consultations with the DPs if there were implausibility or technical problems. For bigger plausibility problems, single new transfers of “corrected” data packages were also scheduled. The use of self-generated codes which are not defined in international diagnostic classifications in some cancer registries is an example to show that it makes sense to carry out basic validity checks centrally.

The basic principle of this project, namely that the cohort data of the single DPs were sent to a data centre (WP1) instead of three outcome-related WPs, proved successful: WP1 was responsible for carrying out validity checks of all variables which did not refer to the outcome relevant characteristics. Otherwise, each WP would have needed to come up with and could have realised its own solution, and the data sets would not have been comparable. So all WPs profited from this procedure. The WPs with additional case–control designs had to set up further specific procedures for additional case–control-related treatment data, which were collected by WP3 and WP4 separately.

However, in general it was the responsibility of the respective WP leaders with their specific know-how to decide upon the outcome-specific variables (e.g., to decide which events were ultimately classified as cardiac events). While inquiries to the DPs were carried out solely via WP1 in the beginning, implausibility in outcome-specific variables were arranged to be clarified directly with the responsible WP leaders for the remainder of the project duration. Within the scope of the case–control study conducted, DPs had to be contacted on the part of WP3 and WP4 (e.g., for assigning controls to cases or for providing therapy data for cases and controls which had not been provided for in the superordinate data set). Due to the amalgamation of the data by a central office and the plausibility checks carried out by these two levels, we can assume high data quality.

Despite the basically unambiguous rules, a number of obstacles occurred, which required complex solutions. These solutions were necessary in order to generate a harmonised, large, and meaningful cohort. Basically, cancer registries are dynamic data sources, in which older data may be modified (subsequent changes, e.g., of diagnosis or age can be seen from time to time) and follow-up information becomes more current the longer the follow-up duration. Therefore, it is recommended that the DPs freeze their data on a specified day and provide them for the overall project. This was, however, hard to communicate, and some DPs kept transferring modified data sets to WP1. This is acceptable in some degree if this leads to a considerably improved data quality. However, marginal changes should not result in new update deliveries. It proved to be difficult to find the right balance.

Limitations of the assembling of this huge retrospective European cohort are that DPs were not always able to provide data as specified in the call-for-data; instead, individual arrangements concerning the data delivery and an adjustment of the central WP1 data base to individual import strategies became necessary. In the end, an individual handling for almost each DP was necessary. This caused temporal delays and the risk was real that some outcome-related WPs would fall behind; as a result, some DPs delivered their data prematurely and multiple times via WP1 to WP leaders, even though data entry and data processing had not been completed. For this reason, many more data updates than intended had to be accepted. The following example demonstrates the complexity: One DP provided 20 data updates altogether, and one WP received 24 data transfers from WP1. In principle, updates were planned only as an exception (step 5 in Fig. 1), and only one single data transfer from WP1 to the respective outcome-related WPs was planned (step 7). In addition, the progress of the work packages went in parallel. However, this could be balanced and compensated by WP1, while three independent, parallel work packages would have been hard to coordinate. Some DPs did not provide data for all three outcomes. In part, this was planned from the beginning (e.g., no cardiac events from the Nordic countries), in part, it became apparent only during the project duration that data could not be provided (e.g. mortality data from Norway are in general available, but could not be provided within the scope of this project). The duration of observation differed for the single events among the data sets of some DPs (e.g., longer duration for cardiac events than for the occurrence of second tumours).

In order to make the ultimate cohort centrally available after the end of the project, the data bases of WP3-5 will finally be transferred to WP1 again. WP1 will store the data and make them available for future projects, should the occasion arise. The cohorts finally analysed in the work packages (e.g. as basis for case–control studies) will differ from the cohort described here due to WP-specific eligibility criteria. Nevertheless, the PCSF cohort described is the basis for all analyses to be carried out in PanCare-SurFup as well as for projects going beyond the end of the project.

In a consortium like this one, progress largely depends on iron discipline and rigour with respect to the common rules for project management. All partners must follow the specifications of the consortium (deadlines, agreements, definitions). As a basic principle, a transparent, prompt, and problem-oriented communication is a necessary basis for the success of such a complex project. Within the course of the project, these processes proceeded more and more smoothly.

Limitations of the consortium are that assembling a huge cohort like this takes a lot of time and this took in the end much longer than anticipated from the beginning. PCSF applied for and was granted a 1-year no-cost extension. Data assembled many decades ago were difficult to collect in some countries. Data management, databases, and data differed from country to country mainly due to different ways of collecting the cancer data and the outcomes, requiring major efforts to make the data homogeneous and comparable.

There are some lessons learned and ways to overcome problems during the implementation of such a diverse cohort to be composed by bringing together very different data sets from different countries. It is strongly recommended that one central institution is installed for doing all the work regarding harmonization, standardization and communication. An iron discipline has to be conformed as well as rigour with respect to the common rules for project management. A transparent, prompt and problem-oriented communication is needed, too. The involved parties should find the right balance between being adamant about standardized procedures while on the other hand considering individual country-specific and data provider-specific framework conditions. Regarding the practical approach data providers should freeze their relevant data set on a specific day and avoid updates with only marginal modifications. The ultimate cohorts should be made centrally available at the end of the project by each work package leader and should have backups to enable sustainability and long-lasting data security.

Benefits of the consortium assembling late effects data is that rare late effects detected in more countries can be pooled and this might lead to new strategies for identifying ways to treat late effects and reach best clinical follow up. The assembled cohort is the largest cohort in Europe and under a handful others under the largest worldwide. Amalgamations of this kind enable analyses which would not have been possible because the diseases are so rare. The scientific legacy produced by PanCareSurFup is available for maintenance, update, and future use in accordance with the regulations set up after the official funding end of the project. Therefore, a PanCareSurFup Sustainability, Publication and Authorship Policy has been developed, which includes that requests from outside investigators for use of the PCSF data will be welcome at least 5 years from the end of the study. The final datasets from each work package of PanCareSurFup are stored at the original work package leader’s institution. Back-ups of all data are stored at defined other institutions.

PanCareSurFup succeeded in compiling the largest and in itself homogeneous cohort of children with cancer during childhood and adolescence through the close cooperation of many European countries and by establishing a work package solely for the harmonisation of heterogeneous data sources. We can expect high quality results analysing this large data set with respect to the three outcomes in PanCareSurFup. The resulting data set provides an excellent opportunity to compare outcomes of patients diagnosed over seven decades.

Depending on the national situation per data provider, informed consent was obtained from all individual participants included in the study, or the data collection was done under national law. All data providers obtained ethical approval or approval from the relevant national body, and PanCareSurFup was supervised by the PCSF Ethical and Scientific Advisory Board.
